# DNase-capture reveals differential transcription factor binding modalities

**DOI:** 10.1371/journal.pone.0187046

**Published:** 2017-12-28

**Authors:** Daniel Kang, Richard Sherwood, Amira Barkal, Tatsunori Hashimoto, Logan Engstrom, David Gifford

**Affiliations:** 1 Computer Science and Artificial Intelligence Laboratory, Massachusetts Institute of Technology, Cambridge, Massachusetts, United States of America; 2 Division of Genetics, Department of Medicine, Brigham and Women’s Hospital and Harvard Medical School, Boston, Massachusetts, United States of America; Yeshiva University Albert Einstein College of Medicine, UNITED STATES

## Abstract

We describe DNase-capture, an assay that increases the analytical resolution of DNase-seq by focusing its sequencing phase on selected genomic regions. We introduce a new method to compensate for capture bias called BaseNormal that allows for accurate recovery of transcription factor protection profiles from DNase-capture data. We show that these normalized data allow for nuanced detection of transcription factor binding heterogeneity with as few as dozens of sites.

## Introduction

DNase-seq is a genomic technique that profiles chromatin accessibility. In DNase-seq, intact nuclei are exposed to the DNase I enzyme. The DNase I enzyme is sterically hindered by compacted chromatin, tightly wound histones, and bound transcription factors and thus produces DNA cuts primarily in the accessible genome. These DNase I cuts result in a pool of molecules that represents the accessible part of the genome that is then sequenced [1]. The resulting DNase-seq data have been used to identify active portions of the genome [1], prioritize genomic variants from genome wide association studies [2], and estimate the transcription factor occupancy of the genome in a single experiment [3,4]. However, DNase-seq is limited by the current cost of sequencing, resulting in inadequate read counts and thus incomplete biological discovery.

Here we introduce DNase-capture, a method that focuses DNase-seq analysis on selected genomic regions, resulting in a substantial increase in region-specific sequencing coverage. The consequential increased analytical resolution allows for the nuanced detection of transcription factor binding modes that are associated with differences in the bound genomic sequences. We also introduce BaseNormal, a base-pair resolution approach to normalization that corrects biases from DNase-capture data. Previously, Stergachis et al. [5] introduced a DNase-capture approach without normalization.

## Results and discussion

**Fig 1:**
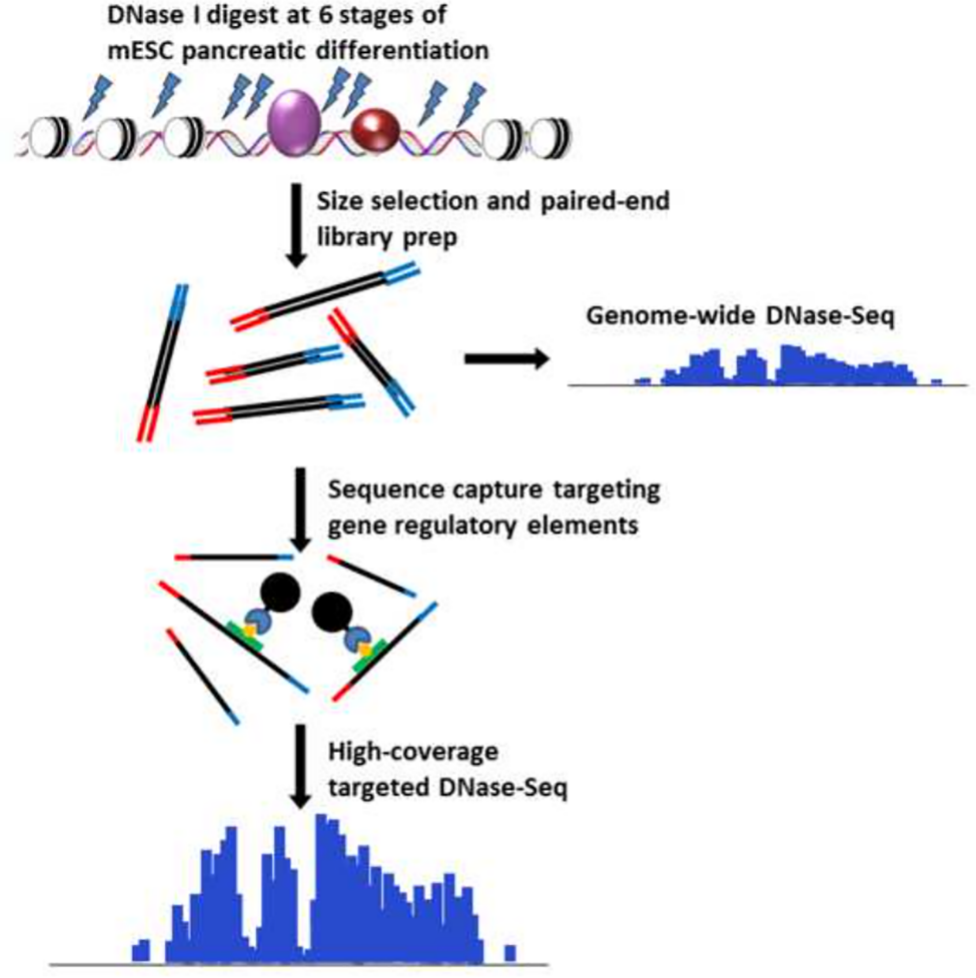
DNase-capture protocol. This figure shows the stages of the DNase-capture protocol.

DNase-capture extends DNase-seq using an analog of an exome capture protocol [6]. In DNase-capture, a DNase-seq library is mixed with biotin-tagged bait RNA sequences that are complementary to regions of interest. In a hybridization reaction, target DNA is captured away from background DNA through streptavidin bead binding, and the captured target DNA is sequenced (Fig 1). To determine whether capture can increase the resolution of DNase-seq, we designed a DNase-capture bait library targeting 91 genomic loci spanning 300 kb of genomic space and using 18,000 bait probes. We define *tiling density* as the number of baits that overlap each base in a design, and thus at a 2X tiling density each base is covered by two distinct baits. Because the transcription factor CTCF is known to leave a strong, spatially stereotyped DNase I footprint when bound [7], we tiled baits at fifty 1 kb regions centered on known CTCF binding sites. The fifty regions were tiled at densities of 2X, 3X, 4X, 6X, and 8X (10 loci were tiled at each density) to determine if tiling density affects capture strength or fidelity. We additionally tiled at 3X density 41 genomic loci encompassing regulatory regions near genes with dynamic expression during mouse embryonic stem cell (mESC) differentiation. Baits in highly repetitive regions were eliminated to prevent non-specific capture. We then performed hybrid capture on previously sequenced DNase-seq libraries from mESC [3].

**Fig 2:**
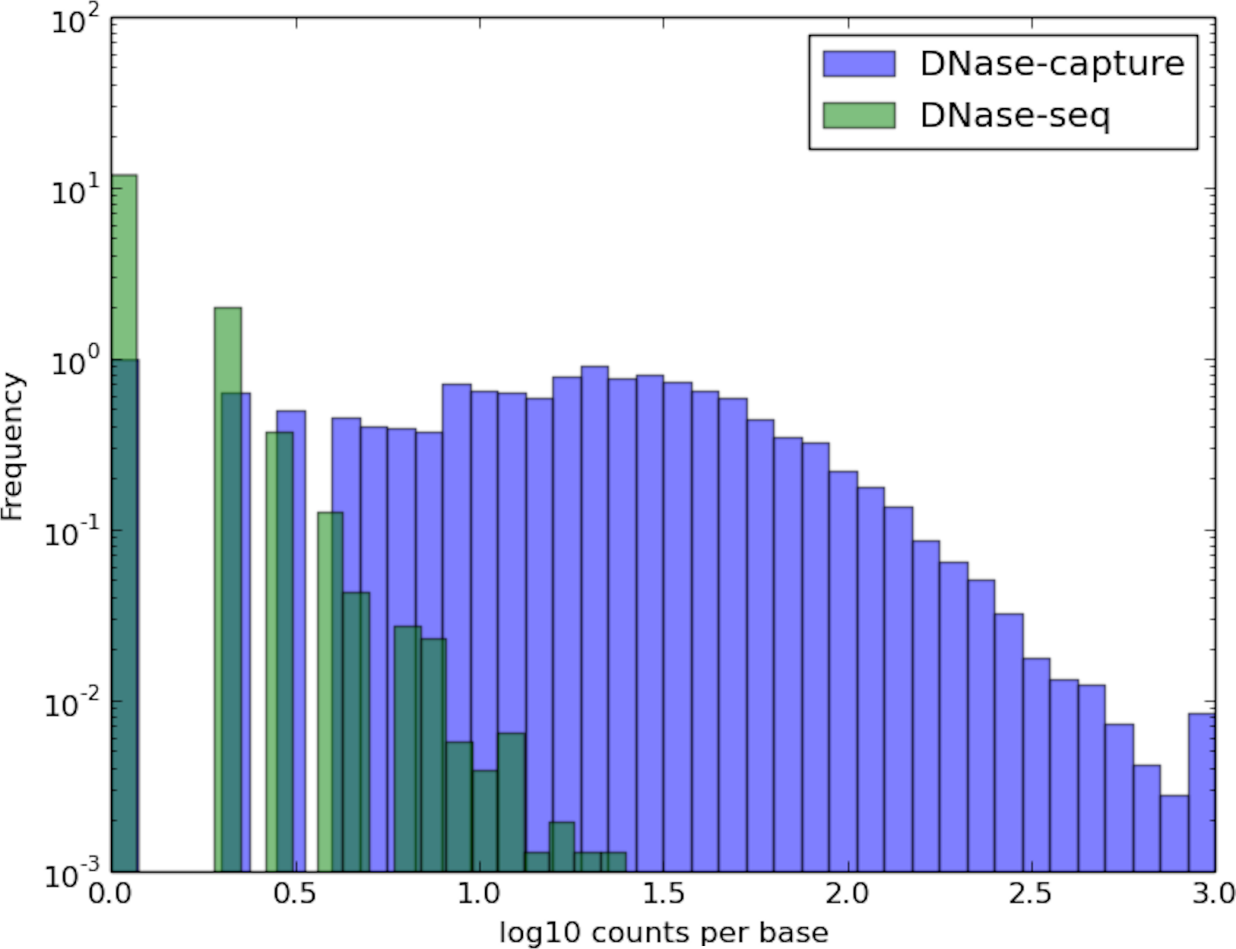
DNase-capture has more reads per base. Reads per base, excluding bases with no reads, for the forward reads. The x- and y-axes are in log 10 scale. The DNase-capture reads were truncated at 1000 reads for visualization purposes. Reads from all tiling densities were aggregated in this plot.

**Fig 3:**
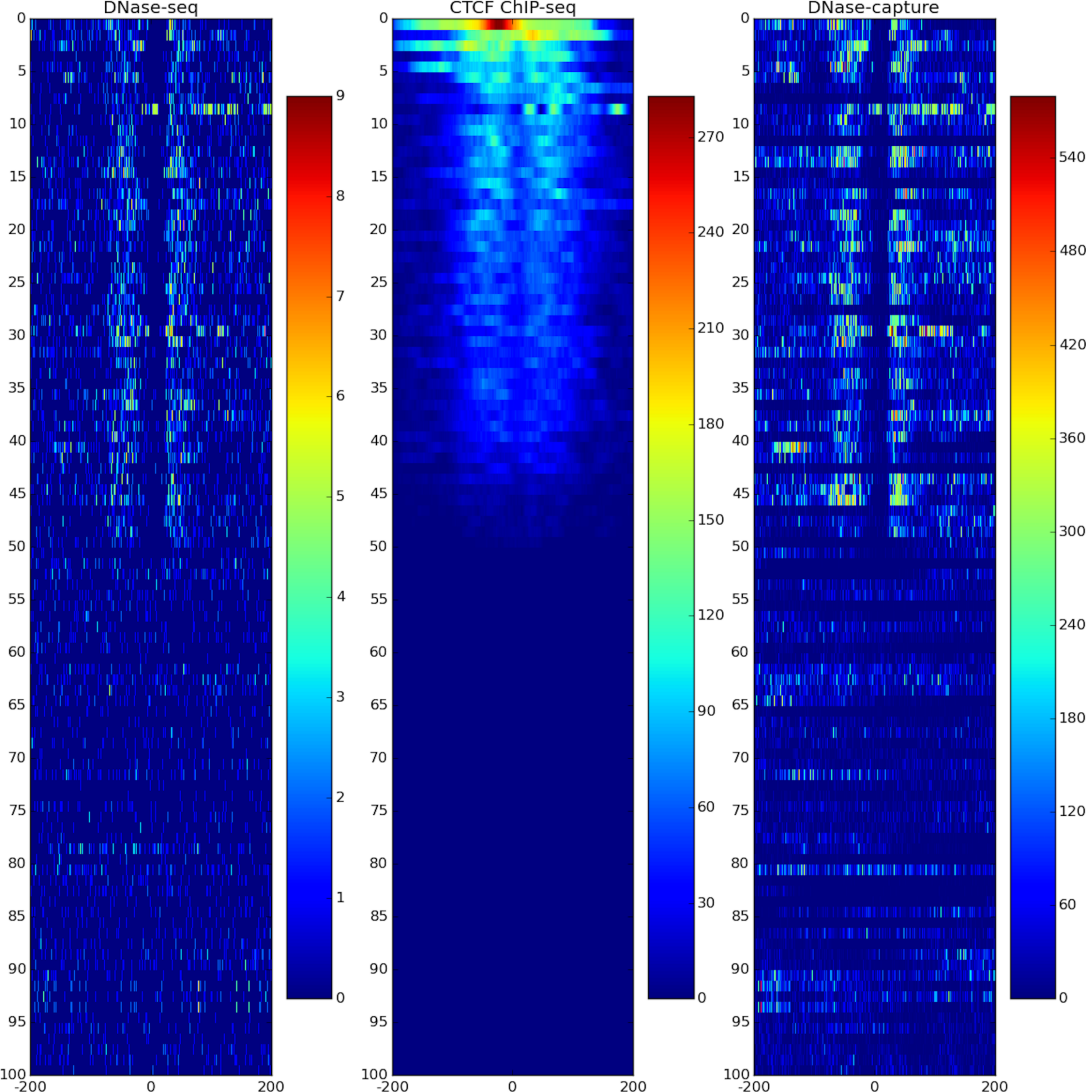
DNase-capture clarifies CTCF DNase profiles. DNase-seq (left), DNase-capture (right), and CTCF ChIP-seq data (center) CTCF motif hits ranked by the number of CTCF ChIP-seq reads in the region. Many DNase-capture profiles are clearer than their DNase-seq counterparts. The bottom half shows CTCF motif matches, but are not called by GEM. The data pre-correction was used here. The legend in the figure shows the motif hit counts scale, with dark red representing more hits, and dark blue representing fewer hits. Note that the footprints represented in lines 4, 34, 44, and 45 are significantly better defined in the DNase-capture data than in the DNase-seq data.

**Fig 4:**
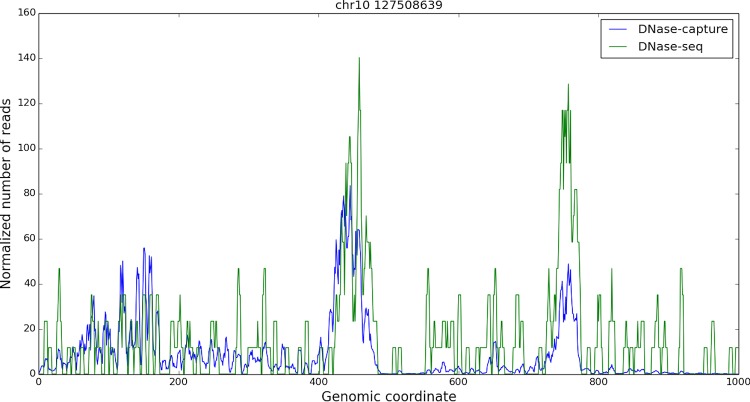
DNase-seq and DNase-capture appear concordant. A one kilobase capture region in chromosome 10, starting at base 127508639. The reads are smoothed in a 5bp window, and only the forward reads are shown for visualization purpose. The DNase-capture reads counts were scaled so they could be shown on the same plot as the DNase-seq reads. The data pre-correction was used here.

**Fig 5:**
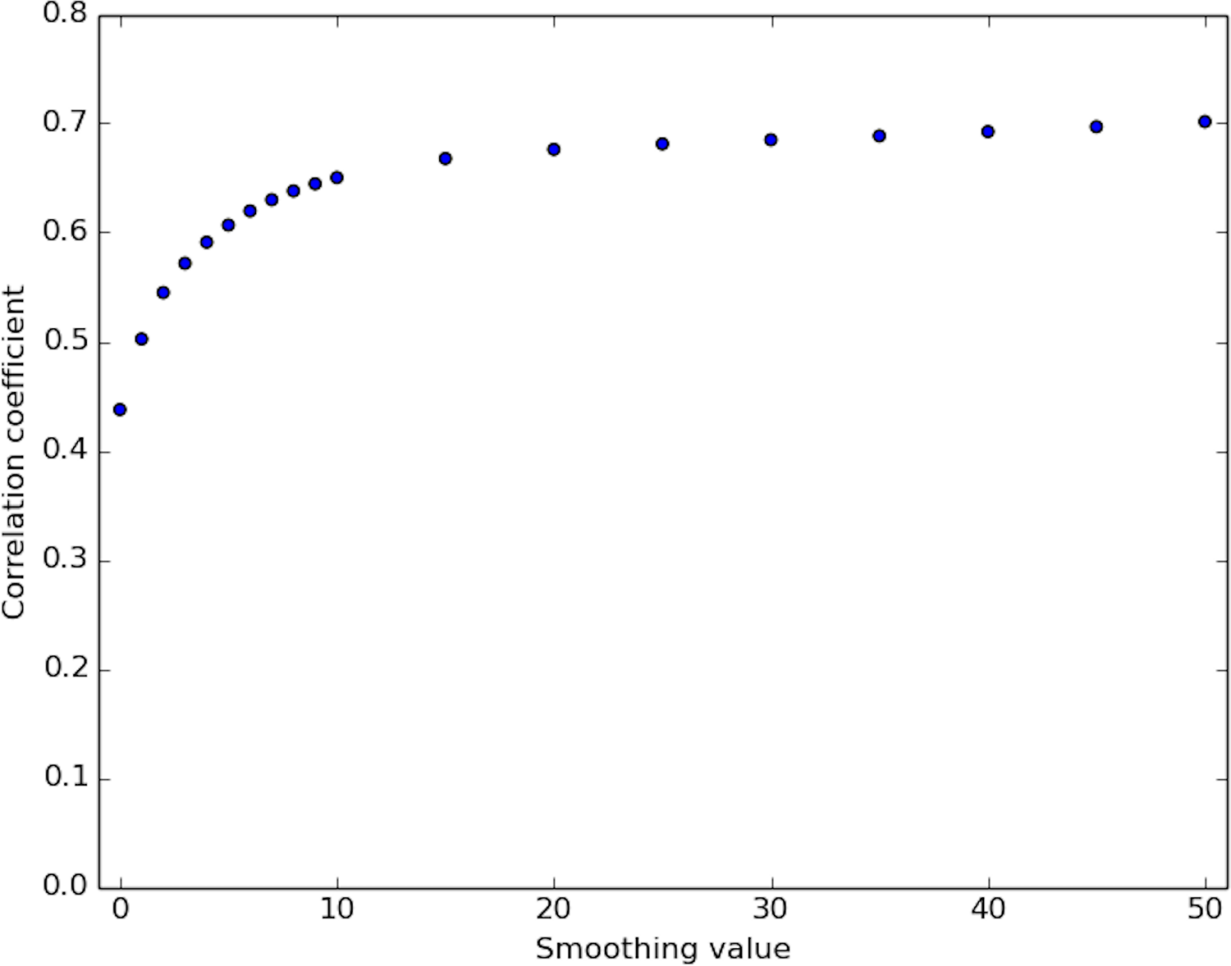
Correlation of DNase-capture and DNase-seq. Correlation coefficient values of the DNase-capture vs the DNase-seq data at various smoothing window sizes in the pre-selected genomic loci. Data pre-normalization was used.

DNase-capture substantially and consistently improves sequencing coverage of targeted regions, with over a 145-fold enrichment in read counts compared to experiments without enrichment with the same overall sequencing depth. DNase-capture consistently shows more reads per base on targeted regions compared to regular DNase-seq (Fig 2). We hypothesized that the increased coverage of DNase-capture would allow for qualitative improvements in observed DNase accessibility profiles [3]. To test our hypothesis, we examined the difference in data from capture regions that had CTCF binding events and motifs as a positive set and CTCF motifs with no binding events as a negative set to ensure DNase-capture did not introduce artifacts. We observed that stereotypical CTCF DNase accessibility profiles containing a center footprint underlying the motif and flanking regions of increased hypersensitivity are significantly better defined in the DNase-capture data at individual binding site instances than in the DNase-seq data (Fig 3). Lines 4, 34, 44, and 45 in particular in Fig 3 represent such footprints. The observed DNase-capture signal is largely concordant with regular DNase-seq signal in matched regions (Fig 4, Fig 5).

**Fig 6:**
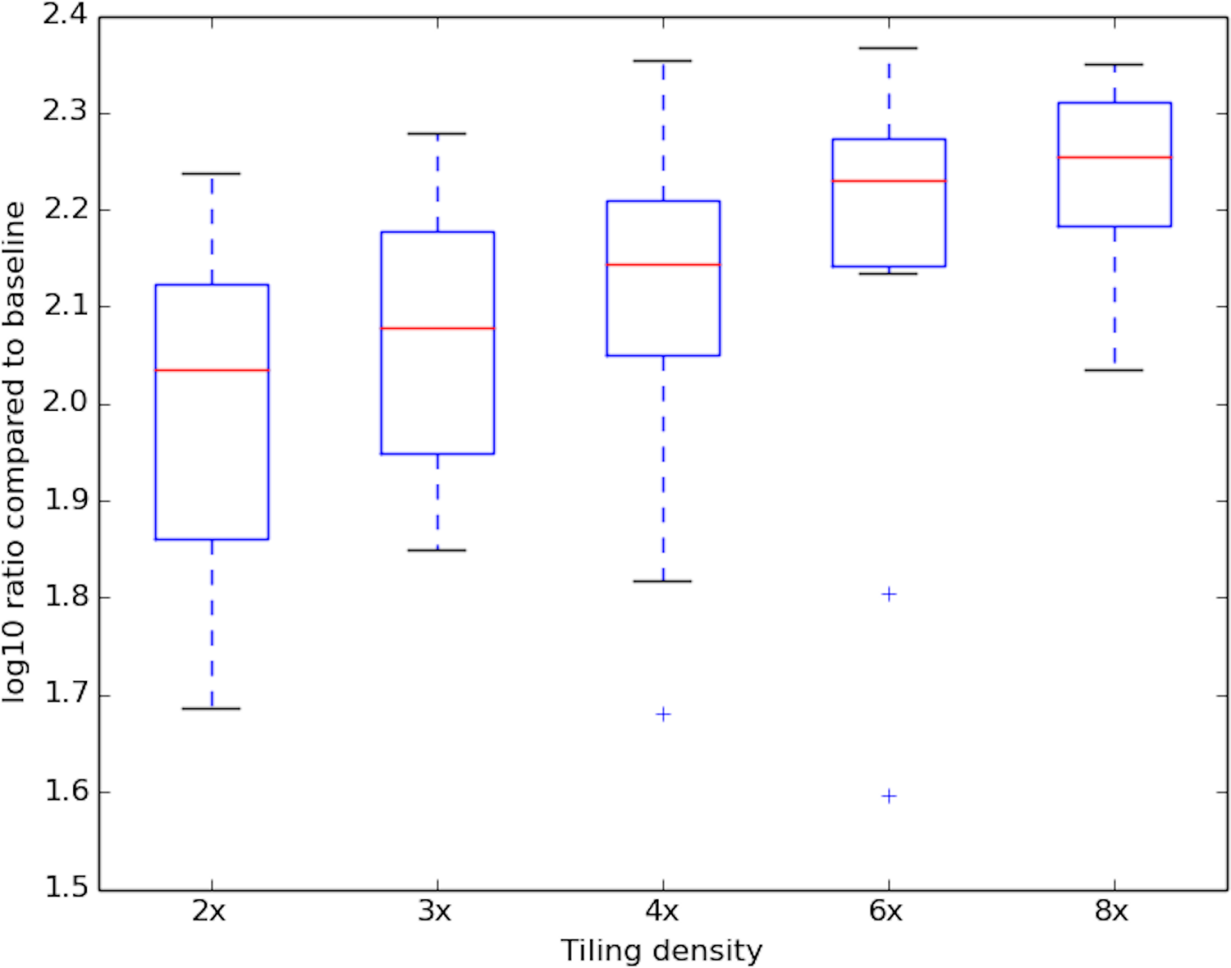
Increased tiling density produces more coverage. Log enrichment of the DNase-capture reads at a given tiling density compared to the number of DNase-seq reads over the same regions. Data pre-normalization data was used.

With the improved resolution of DNase-capture data comes coverage bias that must be computationally normalized for proper analysis. Coverage bias results from different effective tiling densities at different genomic regions, the elimination of certain bait sequences for lack of specificity, and bait-specific capture bias. The effect of tiling density on observed DNase-capture read count is shown in Fig 6. Since DNase-seq analysis depends upon base-pair resolution DNase profiles, existing segmentation-based capture normalization techniques developed for copy number variation and SNP detection [8,9] are insufficient. Existing normalization methods for exome capture [8,9] divide the genome into discrete segments that are independently normalized and applying such methods would introduce artifacts when DNase-seq profiles overlap a segment boundary.

**Fig 7:**
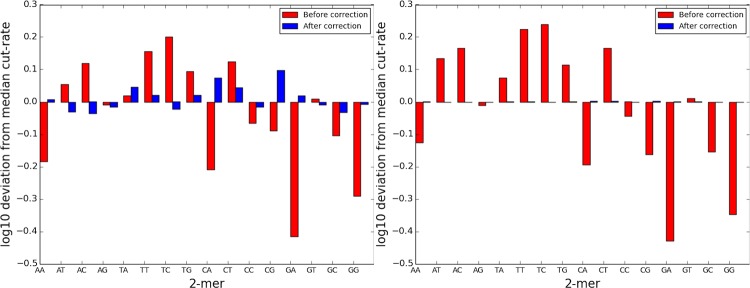
Reduction of two-mer bias in DNase-seq data. Histogram of the median centered two-mer bias of the regular DNase-seq and the correction, after applying the 6-mer correction. The left panel is the DNase-seq data and the right panel is the DNase-capture data. 2-mer bias is reduced after the correction.

**Fig 8:**
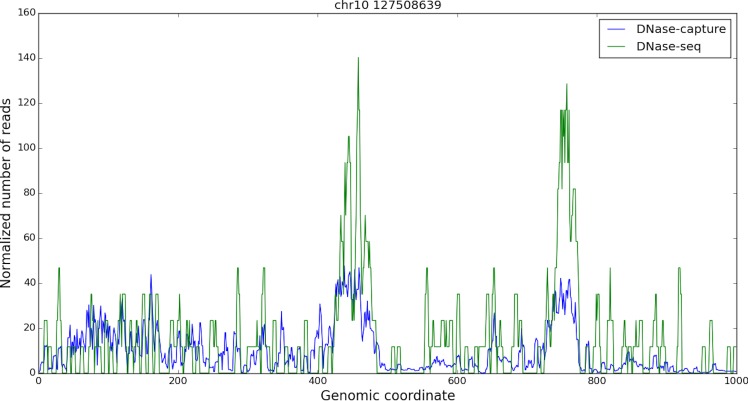
Corrected DNase-capture and DNase-seq appear concordant. A one kilobase capture region in chromosome 10, starting at base 127508639. The reads are smoothed in a 5bp window, and only the forward reads are shown for visualization purpose. The DNase-capture reads counts were scaled so they could be shown on the same plot as the DNase-seq reads. Data normalization was used here.

**Fig 9:**
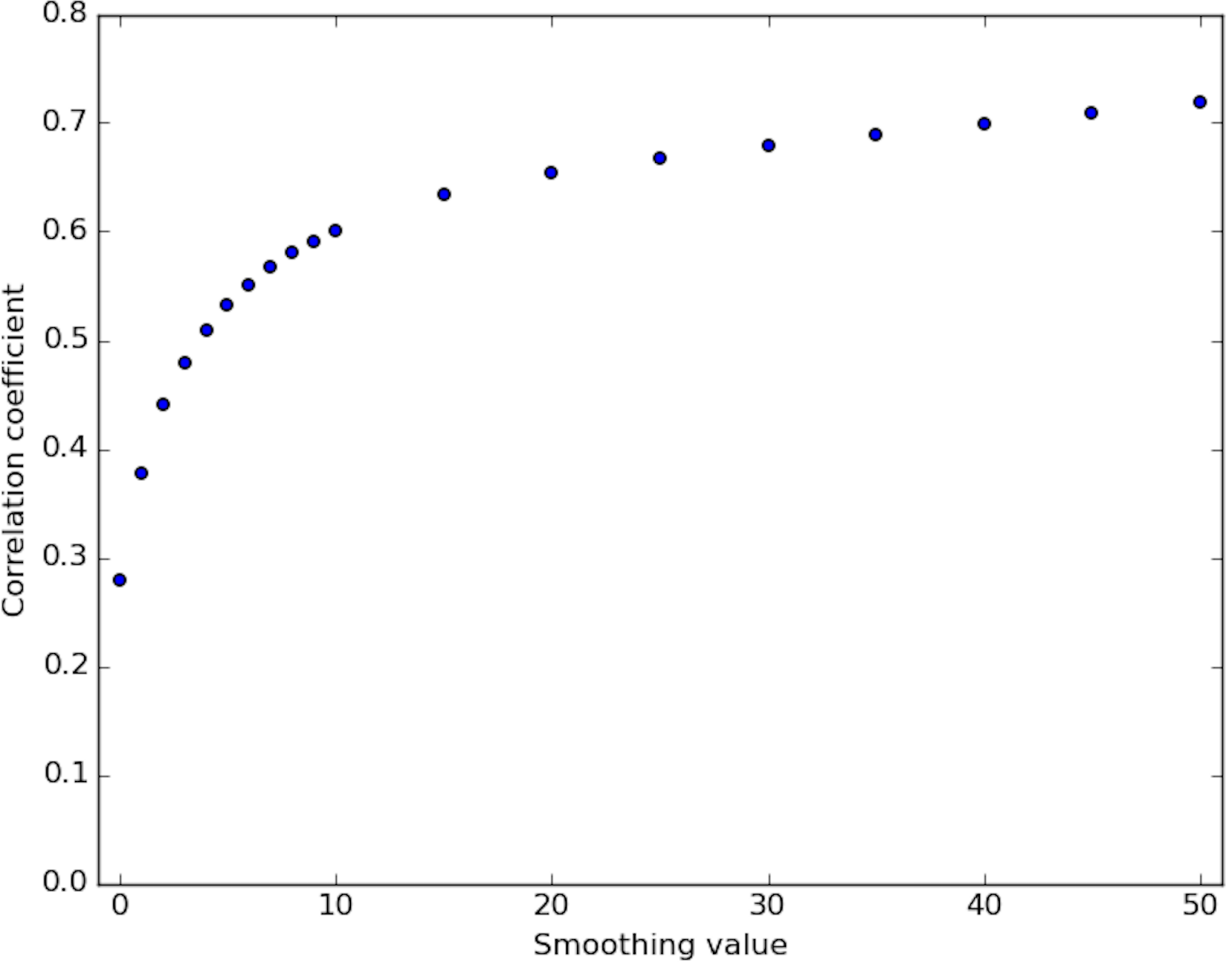
Correlation of corrected DNase-capture and DNase-seq. Correlation coefficient values of the DNase-capture vs the DNase-seq data at various smoothing window sizes in the pre-selected genomic loci. Data normalization was used here.

BaseNormal is a base-pair resolution approach to normalization that corrects DNase I cut bias [10,11], missing bait bias, tiling density bias, genomic cut-rate bias, and read tower bias. Read towers result from a small number of bases that have abnormally high DNase-capture read counts. We perform base-level correction in four steps. First, BaseNormal uses linear regression with DNase-capture reads as the independent variable and DNase-seq reads as the dependent variable. The predicted values from the linear regression represent bias controlled estimates for accessibility. The linear regression is performed on (1) DNase-capture reads +/- 15bp of the base to be predicted, and (2) genomic DNase-capture reads +/- 15bp of the base to be predicted (the genomic DNase-capture reads are used to control for bait affinity). We use a truncation indicator based on the genomic DNase-capture reads to control for read tower biases. The truncation indicator is set for any base where the number of genomic capture reads were above the 95th percentile of the genomic capture reads. Second, we correct for DNase-I cut bias [10,11] by normalizing the number of reads at each 6-mer to the average number of reads starting at the same 6-mer in a prior naked DNase-I digestion experiment [11]. The effects of correcting for sequence bias on the DNase-cut frequency for various dinucleotides are shown (Fig 7). We hypothesize that the substantial increase in sequencing coverage increased bias reduction in DNase-capture data in the capture regions. Third, since certain highly repetitive baits were not synthesized, bases within +/- 10bp of these baits were not considered in the regression. Fourth, to account for regions with different tiling densities, BaseNormal performs a region-by-region adjustment of the average DNase-capture reads per base, adjusting them to be proportional to the average reads per base from a non-capture DNase-seq experiment. This normalization adjusts all of the read counts in a given region identically. The corrected DNase-capture signal is largely concordant with the regular DNase-seq signal (Fig 8, Fig 9).

We posited that if we clustered the profiles of accessibility data around a factor’s binding site we would discover distinct classes of DNase profiles that reflect distinct binding modes for certain factors. To explore this idea, we identified CTCF binding sites in the capture regions from mouse ChIP-seq datasets using GEM [12]. We excluded binding events without strong motifs so that binding sites could be aligned at the base pair level, and we excluded events within 200bp of a capture region edge. Each binding site was identified with a transcription factor specific DNase-capture profile, and we trained a Bernoulli naïve Bayes classifier on the individual bases surrounding a binding site to predict the DNase-capture profile cluster of each binding event. We measured the classifier’s performance by area under the receiver operating curve (AUROC) using leave-one-out cross validation.

**Fig 10:**
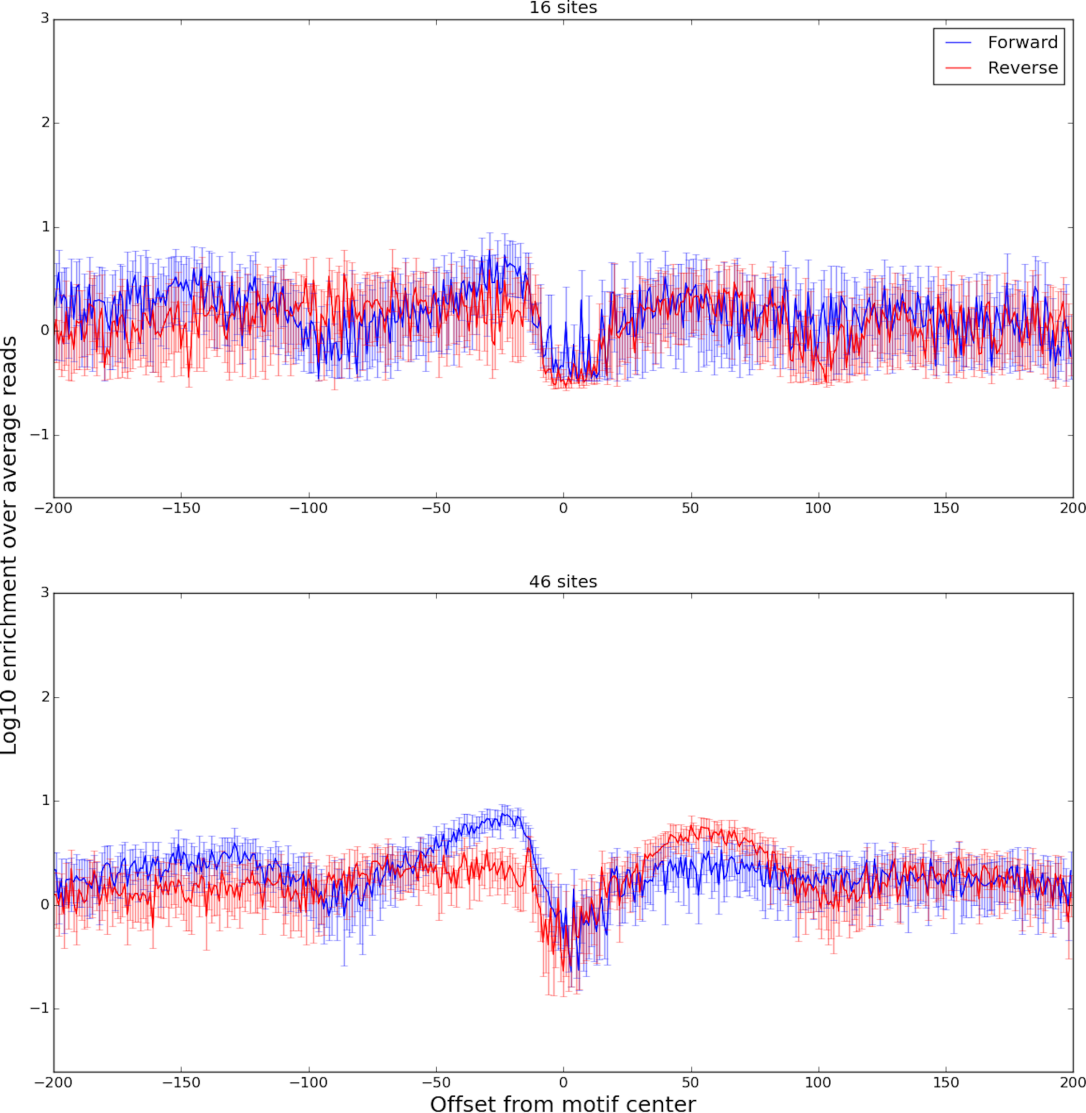
Aggregate plots of two DNase-capture CTCF clusters. The plots show enrichment over the DNase-capture baseline. The bottom panel shows a normal accessibility profile of a transcription factor, with a characteristic dip in the center and a rise on both sides. The top panel shows a novel CTCF accessibility profile, where the left side has the characteristic difference between the forward and reverse strands, but the right side does not. The 95% confidence bounds are calculated through bootstrapping.

**Fig 11:**
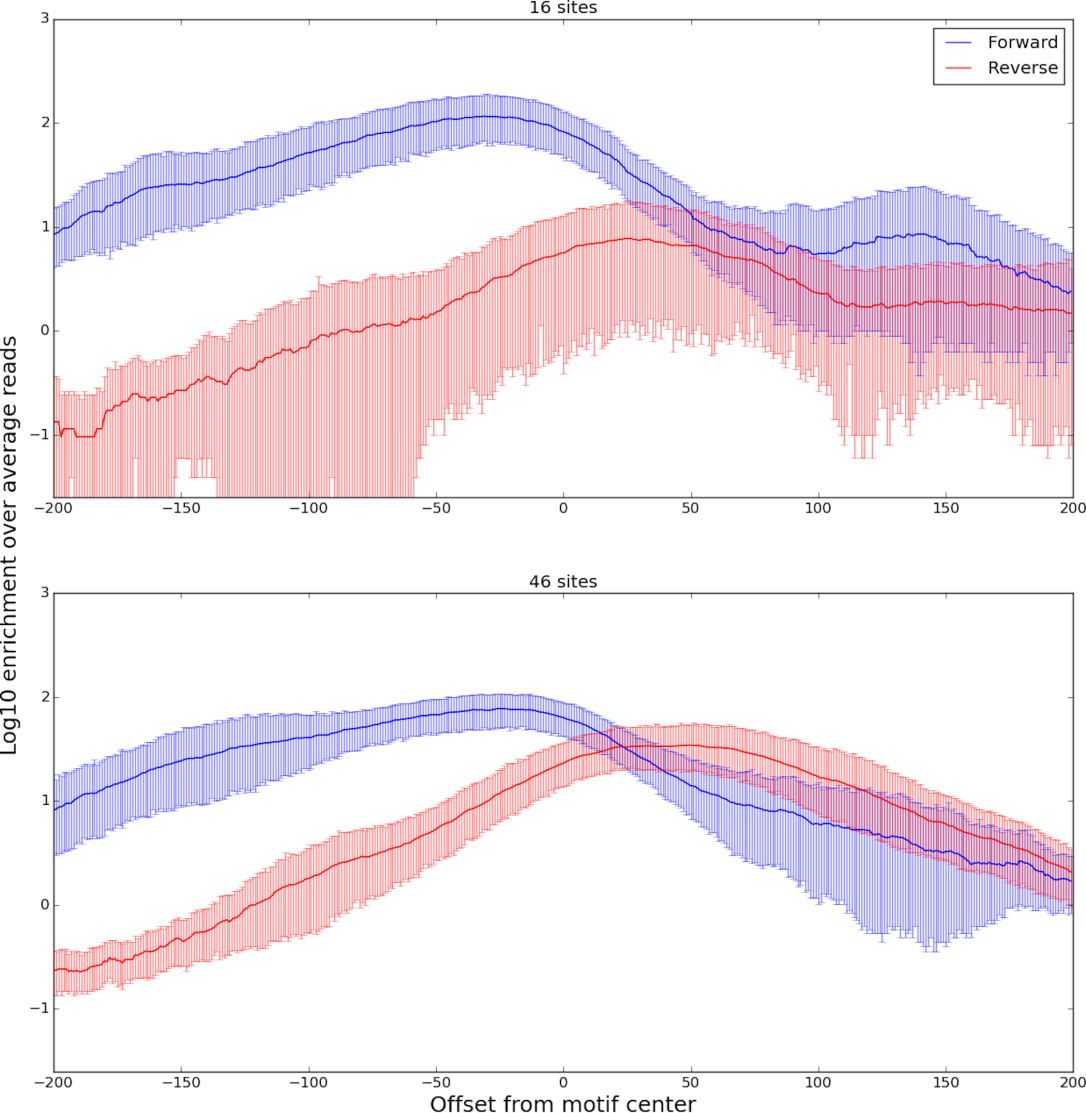
Aggregate plots of two ChIP-seq CTCF clusters. The plots show raw ChIP-seq read counts over the two CTCF clusters. The ChIP-seq profiles are consistent with the DNase-seq profiles of Fig 10.

We found that the DNase-capture profiles of 62 motif present CTCF binding events clustered into two different DNase profiles that represent different binding modes (Fig 10). Our classifier was able to predict the membership of a binding event in its cluster based upon genome sequence with an AUROC of 0.85 with a p-value of 0.049 (Supplementary Methods). The classifier revealed two distinct CTCF binding modes based on the 5’ sequence components of its CC(C/A)(C/T)C central motif. Prior work has shown that CTCF binding was disrupted [13] when the 5’ CCAC binding site sequence was changed to ATAT. Consistent with this observation we found that the 5’ flanking motif sequence was the most important feature for predicting a site’s DNase profile cluster (Fig 10). Consistent patterns were found in the ChIP-seq profiles for the same clusters (Fig 11). We hypothesize that small changes in the flanking sequence affect binding affinity, which in turn is inversely related to the number of DNase-seq reads observed at that location.

## Conclusion

DNase-capture followed by BaseNormal normalization offers a high-resolution view of transcription factor binding at individual sites. This increased resolution allows discovery of distinct sequence-specific binding modalities by individual TFs.

## Supporting information

S1 Text Supplementary materials and methods. The text includes supplemental information on the protocol, DNA sequence processing, accession codes, BaseNormal correction, ChIP-seq peak calling, and AUROC p-value computation.
(PDF)
