# Supplementary material for: DNase-capture reveals differential transcription factor binding modalities
Source: PLoS One. 2017 Dec 28;12(12):e0187046. doi: 10.1371/journal.pone.0187046 (PMC5746236; doi:10.1371/journal.pone.0187046)
Supplement: S1 Text — The text includes supplemental information on the protocol, DNA sequence processing, accession codes, BaseNormal correction, ChIP-seq peak calling, and AUROC p-value computation. (PDF) [file pone.0187046.s001.pdf]

Supplementary Materials and Methods

Protocol

DNase-seq sample collection and library preparation were performed according to Sherwood et al 2014 [3]. Briefly, 129P2/OlaHsd mouse embryonic stem cells were harvested at different stages of pancreatic differentiation and subjected to DNaseI enzyme treatment followed by collection of 50-125 or 175-400 bp hypersensitive DNA. Illumina adapters were added according to standard Illumina protocols at the MIT BioMicroCenter. Libraries used in this study are the same as were used in Sherwood et al 2014 [3].

Sequence capture biotinylated baits were synthesized by Mycroarray, and sequence capture of the DNase-seq libraries was performed according to Mycroarray's protocol, which includes 15 cycles of PCR amplification of captured DNA regions. A list of sequence capture baits is included. Sequence capture libraries were then sequenced using Illumina HiSeq by the MIT BioMicroCenter.

Accession codes

GSE53776

GSM912896

GSM912908

GSE106217

DNA sequence processing

All raw DNA sequence data was aligned with BWA using settings bwa version 0.6.2 with the default settings and 8 threads [1]. Raw sequence data was processed as single-ended reads. DNase-seq and DNase-capture reads were filtered so only reads with a mapping quality of 20

were retained. Coverage at a base was calculated using only the outermost coordinate of a read. DNase-seq and DNase-capture replicates were kept separate.

BaseNormal DNase-capture normalization

BaseNormal correction is described is the order in which the corrections are applied.

Previous studies have shown that the sequence affinity of DNase-I contributes substantially towards the bias of DNase-seq and that this bias can be characterized by 6-mers. To correct for the sequence affinity of DNase-I, we used the 6-mer bias table computed by cut rates in a naked DNA digestion [11]. The DNase-seq and DNase-capture data was then adjusted at a base by base level by the table to correct for the 6-mer cut bias, by dividing the number of reads in the DNase-seq and DNase-capture data by the value of the table for the given 6-mer.

Different tiling densities were used in different genomic regions, and this resulted in more reads in higher density genomic regions. To correct for the tiling density bias, we normalized the expected counts for each tiling density as a preprocessing step. We calculated the average number of reads in each tiling density and normalized the reads so that each tiling density had the same number of expected reads.

Finally we control for other bias effects arising from the DNase-capture protocol and the effect of read towers by modeling the DNase-seq reads as a linear combination of DNase-capture reads, DNase-capture genomic control reads, and two indicators for read towers, one for each dataset. The fitted values (the predicted y_i as defined below) are the bias controlled estimates for accessibility we use. The features used in the linear regression for given experiment are its observed DNase-capture reads, DNase-capture genomic control reads, and an indicator for the genomic and DNase-capture reads if they were above the 95th percentile, each with a +/- 15bp window. Formally, denote the reads from the DNase-capture genomic control at base i to be g_i

and the reads for a given DNase-capture experiment to be c_i, both truncated at their respective 95th percentiles. Let I_{g_i} and I_{c_i} to be indicators that g_i and c_i were truncated respectively. Let y_i be the reads of the DNase-seq experiments at base i. Finally, let $\beta_{j,k}$ be the coefficients of regression. Then, the regression is in the form

$$y_i = \sum_{j=-15}^{15} \beta_{j,1} g_{i+j} + \beta_{j,2} c_{i+j} + \beta_{j,3} I_{g_i} + \beta_{j,4} I_{c_i}$$

The regression was done using Vowpal-Wabbit [2], a fast, open-source regressor. Default options were used with Vowpal-Wabbit, except two passes were used in the learning step. The default loss function is L2.

ChIP-seq peak calling

Analogously to how DNA sequence was processed, we realigned raw ChIP reads as single-paired reads and the same BWA settings for DNA sequence processing was used.

ChIP-seq peaks were called using the GEM peak caller [3] using the recommended parameters k_min=6, k_max=13, and s=2000000000. ChIP-seq peak calling was done with all the reads, to better learn the ChIP-seq profiles. For analysis, we only used peaks that were called within the capture region and with motifs.

AUROC p-value computation

To compute the p-value of the AUROC, we used a permutation test. Specifically, the class labels for the examples were randomly permuted, and the leave-one-out validation was redone with the permuted labels. The permutation and validation was repeated 1000 times. To compute the p-value, we computed the rank of the true AUROC value of the 1000 trials, divided by 1000, and subtracted the result from 1.

References

1. Li H & Durbin R. Fast and accurate short read alignment with Burrows-Wheeler transform. *Bioinforma. Oxf. Engl.* **25,** 1754–1760 (2009).
2. Agarwal A, Chapelle O, Dudík M & Langford J. A Reliable Effective Terascale Linear Learning System. *J. Mach. Learn. Res.* **15,** 1111–1133 (2014).
3. Guo Y, Mahony S & Gifford D. High Resolution Genome Wide Binding Event Finding and Motif Discovery Reveals Transcription Factor Spatial Binding Constraints. *PLoS Comput Biol* **8,** e1002638 (2012).
